# Proposal of a Method for Transferring High-Quality Scientific Literature Data to Virtual Patient Cases Using Categorical Data Generated by Bernoulli-Distributed Random Values: Development and Prototypical Implementation

**DOI:** 10.2196/43988

**Published:** 2023-03-09

**Authors:** Christian Schmidt, Dorothea Kesztyüs, Martin Haag, Manfred Wilhelm, Tibor Kesztyüs

**Affiliations:** 1 Medical Data Integration Center Department of Medical Informatics University Göttingen Göttingen Germany; 2 GECKO Institute Heilbronn University of Applied Sciences Heilbronn Germany; 3 Department of Mathematics Natural and Economic Sciences Ulm University of Applied Sciences Ulm Germany

**Keywords:** medical education, computer programs and programming, probability, rare diseases, diagnosis, medical literature, automation, automated, virtual patient, simulation, computer based, Bernoulli

## Abstract

**Background:**

Teaching medicine is a complex task because medical teachers are also involved in clinical practice and research and the availability of cases with rare diseases is very restricted. Automatic creation of virtual patient cases would be a great benefit, saving time and providing a wider choice of virtual patient cases for student training.

**Objective:**

This study explored whether the medical literature provides usable quantifiable information on rare diseases. The study implemented a computerized method that simulates basic clinical patient cases utilizing probabilities of symptom occurrence for a disease.

**Methods:**

Medical literature was searched for suitable rare diseases and the required information on the respective probabilities of specific symptoms. We developed a statistical script that delivers basic virtual patient cases with random symptom complexes generated by Bernoulli experiments, according to probabilities reported in the literature. The number of runs and thus the number of patient cases generated are arbitrary.

**Results:**

We illustrated the function of our generator with the exemplary diagnosis “brain abscess” with the related symptoms “headache, mental status change, focal neurologic deficit, fever, seizure, nausea and vomiting, nuchal rigidity, and papilledema” and the respective probabilities from the literature. With a growing number of repetitions of the Bernoulli experiment, the relative frequencies of occurrence increasingly converged with the probabilities from the literature. For example, the relative frequency for headache after 10.000 repetitions was 0.7267 and, after rounding, equaled the mean value of the probability range of 0.73 reported in the literature. The same applied to the other symptoms.

**Conclusions:**

The medical literature provides specific information on characteristics of rare diseases that can be transferred to probabilities. The results of our computerized method suggest that automated creation of virtual patient cases based on these probabilities is possible. With additional information provided in the literature, an extension of the generator can be implemented in further research.

## Introduction

### Background

Education in medicine is a complex constellation of experienced teachers, instructive case studies supported by actual patients when possible, and motivated students. Teachers in medicine have at least two main roles: One role is their clinical practice, and the other role is teaching. In many countries, medical teachers are also expected to conduct research, which requires tight time management to accommodate all 3 roles [1]. However, teaching is complex work, and there are several criteria a teacher has to consider. An elaboration of these criteria can be found in the “seven-component-framework to enhance teaching effectiveness” [2] and include issues such as communication of goals, which is the basis for assessment [3]. Furthermore, especially in medical teaching, there are skills that cannot be taught in the classroom, such as clinical practice [3]. Teachers in medicine need to be experienced because medicine is, in contrast to many other subjects, an experience-based subject. However, experienced medical staff are usually severely time constrained by a variety of patient care tasks, a considerable amount of administrative or documentation duties, and other activities like meetings and organizing [4]. Additionally, increasing clinical obligations, partly due to economic constraints, and the lack of protected time resources (such as times for academic teaching or other nonclinical activities) make it more difficult for clinicians to fulfill academic tasks [5,6]. For teaching purposes, this staff must therefore be considered a limited resource that is not easily available. However, in addition to the severe time constraints on teachers, some other problems hamper the clinical education of students. When a particular disease is to be taught, patients with the corresponding diagnosis are usually not easily available. This applies especially for rare diagnoses. As a result, it may happen that there is a lack of adequate medical practice for medical students [7]. This is aggravated by the fact that the time patients stay in the hospital is reduced. Furthermore, some diagnoses (eg, tick-borne diseases) occur only seasonally and cannot be taught during the whole year [8]. Hence, there may be a gap in the training of especially rare but life-threatening diseases such as babesiosis, brain abscess, botulism, or abdominal aortic aneurysm rupture.

Further challenges in clinical teaching include competing demands where the needs of patients and students can conflict. This is encouraged by the fact that the clinical environment is not “teaching friendly,” as a hospital ward is not an ideal learning platform [9]. There are a lot of skills that cannot be learned in the classroom or from textbooks, as clinical knowledge can be better learned in a clinical setting. This requires a real patient or a patient simulation [3]. Patients play an important role in medical teaching; they can “tell their stories and show physical signs” [9].

Virtual patients as representatives of real patients in a computer-generated world are used as a solution for the gap in sufficient medical practice for medical students [7]. Virtual patients can be implemented in simulated virtual clinical scenarios [10]. They are often used in e-learning environments and are usually based on real patient histories [11]. Other sources for the design and creation of virtual patients are reformatted data from electronic health records, respectively hospital information systems [12]. According to a systematic review, these virtual scenarios are well accepted in the education of medical students [12]. Virtual patient case studies used in teaching have been shown to improve medical student engagement [13]. Furthermore, case-based learning offers a promising method to assist students in learning the vast amount of clinical information, and the integration of virtual patients and cases can improve the effectiveness of education [14,15]. In addition, virtual patient cases offer the possibility of continuing education for physicians, which can be used especially for diagnostic training and medical decision-making [16]

### Objectives

In our preliminary work, we focus on diagnosis, which is seen as one of the most important foundations in the training of future physicians [17]. Virtual patient cases can make an immense contribution here, especially with regard to the rare diseases already mentioned. Currently, virtual patients have to be elaborately created and filled with real patient data by the educator. Because of this, education using manually created virtual patients suffers from exactly the same problem as overall clinical education in medicine: the limited availability of experienced medical staff. To solve this problem, automated creation of complete virtual patients by a computer program is conceivable but is not yet available due to its complexity.

Automated creation of virtual patient cases may offer many advantages. It relieves some burden on medical staff, and, if evidence-based medical literature is used to create the virtual patient data, the quantity and quality of virtual patient cases can be significantly extended by basing their characteristics not on single subjective observations but on a comprehensive and generally agreed-upon medical consensus, available in a written form [18-20].

The accurate, comprehensive, and detailed description of diseases or disease profiles with all associated information forms the basis for automated creation of virtual patient cases. This information can be found in the medical literature, particularly in evidence-based major medical textbooks such as “Harrison’s Principles of Internal Medicine” [19] or “Mandell, Douglas, and Bennett's Principles and Practice of Infectious Diseases” [20]. In order to use information from the textbooks, it must be available not only qualitatively, such as in terms of various symptoms of a disease, but also quantitatively, in the form of data on the frequency of their occurrence in that specific disease. For further detailed information or specifics, also related to pre-existing conditions, concomitant diagnoses, and special population groups, an additional systematic search in medical databases can be considered.

Symptoms play a pivotal role in the diagnostic process because, together with the medical history, they form the basis for further diagnostic examinations like laboratory tests, computed tomography (CT), or magnetic resonance imaging (MRI). The presence of quantitative information regarding a diagnosis allows for random generation of patient cases with diagnosis-specific information. The core of the automated generation is the Bernoulli experiment, which can generate an assignment of diagnosis-specific properties for each patient case based on the quantitative information. In statistics, a random experiment in which there are only 2 possible outcomes (success or failure, or in the case of a symptom, its presence or absence) is defined as a Bernoulli experiment. Bernoulli experiments are also used in other areas of the medical field. Branson and Bind [21] described a framework for randomization testing for clinical trials and observational studies assuming an assignment mechanism that is based on a Bernoulli experiment. The random decision whether a patient receives a drug substance or the placebo can be modeled by a Bernoulli experiment with success probability of *P*=.5. In a simulation of the stroke-free period in at-risk patients with atrial fibrillation, the incidence of stroke was modeled as a Bernoulli experiment. The prediction of the stroke-free duration was used to estimate the risk of stroke in patients with atrial fibrillation [22]. Another application of Bernoulli experiments was reported in a method for modeling conception in fertility studies [23]. However, we could not identify any publications describing implementation of Bernoulli experiments in the context of medical training cases.

In this work, the following questions were investigated and tested for feasibility:

- Does the medical literature contain sufficient data that can be used to extract qualitative and quantitative information about diagnoses and the probabilities of correlated symptoms?
- How can this information be used to create virtual patient cases considering the different characteristics of diagnoses, such as specific occurrence of symptoms?

## Methods

In accordance with the underlying research questions to test the feasibility of our concept as aforementioned, we first examined the literature data and then explored the possibilities of using the basic information obtained from the literature to automatically generate exemplary patient cases. We based our investigation on the example of the rare but life-threatening disease brain abscess, with incidences ranging from 0.4 to 0.9 cases per 100,000 population [24].

### Information Retrieval

To extract evidence-based information about definite diagnoses, we examined which information about diagnoses is given in medical textbooks and how this information is structured. The results revealed that the textbooks contain detailed information about the occurrence of specific symptoms for certain diagnoses that could be used as the basis for the automated and random creation of a template for virtual patient cases [19,20]. As an example, the symptom “fever” is described in 32%-79% of patients diagnosed with “brain abscess,” a very rare condition that must be diagnosed and treated as soon as possible [24]. In addition to the common symptoms, other diagnostic criteria, for instance, specific symptoms related to the location of the brain abscess or specific clinical characteristics regarding certain pathogens, are also provided in the textbooks.

Complementary to the basic, evidence-based information about a specific disease that can be obtained from medical textbooks, we conducted a systematic search for additional or more sophisticated information in the medical literature that may be used in the future to expand our program. To assess this potential for further supplementation of information from medical textbooks, our search focused on symptoms and diagnosis of brain abscesses and was performed in PubMed and Embase. Both databases were searched using specific key words (brain abscess, symptom, diagnosis, epidemiology) and Boolean operators to meet the requirements. The search strategy was then applied without restriction of language or time period.

### Statistical Computing and Programming

The occurrence of a symptom of a single patient case can be modeled with a Bernoulli distribution. For this purpose, a Bernoulli experiment with the probability *p* for the occurrence of this symptom is performed, where *p* is the probability of success (outcome “1”). For example, the coin toss of a fair coin is a Bernoulli experiment with *p*=1/2 [25], and in our example here, a symptom with the probability *p* from the literature is given instead. However, since the data in the literature are always given as a range of the probability of a symptom occurring, a random number is generated from this range for the underlying probability *p* for each single Bernoulli experiment, in order to reflect the distribution and reach the respective variance of real-world data. Mean values were calculated from the given ranges to control the success of the generator. Hence, for each symptom of a case, a Bernoulli experiment is independently done, resulting in a series of Bernoulli experiments for each case (see Table 1). The first experiment in a series relates to symptom 1, the second experiment to symptom 2, and so on. These series are repeated until the desired number of cases is reached. Table 1 illustrates the method, where each row in the table represents 1 case with the associated symptoms.

To achieve this output, a random number generator was implemented in R, the programming language that is part of the free software of the R Foundation for Statistical Computing [26]. Here, we used the version R 3.6.1. To simulate the performance of Bernoulli experiments, the R function “rbinom” requires 3 arguments: (1) number of observations, (2) number of experiments per observation, (3) probability of success [27]. The last argument would be the probability retrieved from the literature [24]. With the help of the function “cbind” [28], after each individual run of the chain of functions, the respective outcomes are linked to each other, resulting in a series that represents the outcomes of the individual experiments with respect to the symptoms for each case (see Table 1).

**Table 1:** Arrangement of the Bernoulli experiments.

Case	Symptom 1 (Bernoulli experiments)	Symptom 2 (Bernoulli experiments)	...	Symptom m (Bernoulli experiments)
1	0	1	...	0
2	1	1	...	1
...	...	...	...	...
N	1	0	...	1

## Results

### Information Retrieval

With the current state of science, it is possible to extract reliable further information on diagnoses, such as the probabilities of the occurrence of various symptoms, from the medical literature. For example, the diagnosis “brain abscess” is described in a medical textbook with the symptoms and respective probabilities depicted in Table 2 [24]. More usable information with regard to our example diagnosis (eg, on gender and age distribution, symptom constellation for diagnosis, and further diagnostic information such as cerebrospinal fluid and blood parameters of infection) can also be found in the medical literature [24,29-31].

We conducted our systematic literature search in October 2022 and retrieved 50 results from PubMed and 60 nonduplicate results from Embase. The review of this literature revealed several cohort and review studies that addressed specific risk factors, symptoms, prognostic factors, changes over time, and population groups. By far, the largest proportion, however, was case reports and case series dealing with specific pathogens, rare causes and complications, or treatment trials.

**Table 2:** Probability of symptoms from the literature for the diagnosis “brain abscess.”

Symptom	Headache	Mental status changes	Focal neurologic deficit	Fever	Seizures	Nausea and vomiting	Nuchal rigidity	Papilledema
Range of probabilities	0.49-0.97	0.28-0.91	0.20-0.66	0.32-0.79	0.13-0.35	0.27-0.85	0.05-0.52	0.09-0.51
Mean value of the range	0.73	0.60	0.43	0.56	0.24	0.56	0.29	0.30

### Statistical Computing and Programming

The probabilities in the literature were provided as a range, so the probability of success of each single Bernoulli experiment was drawn randomly from this range. Finally, all random successes were summed and divided by the number of drawings and are reported as the estimated probabilities in Table 3.

**Figure 1:**
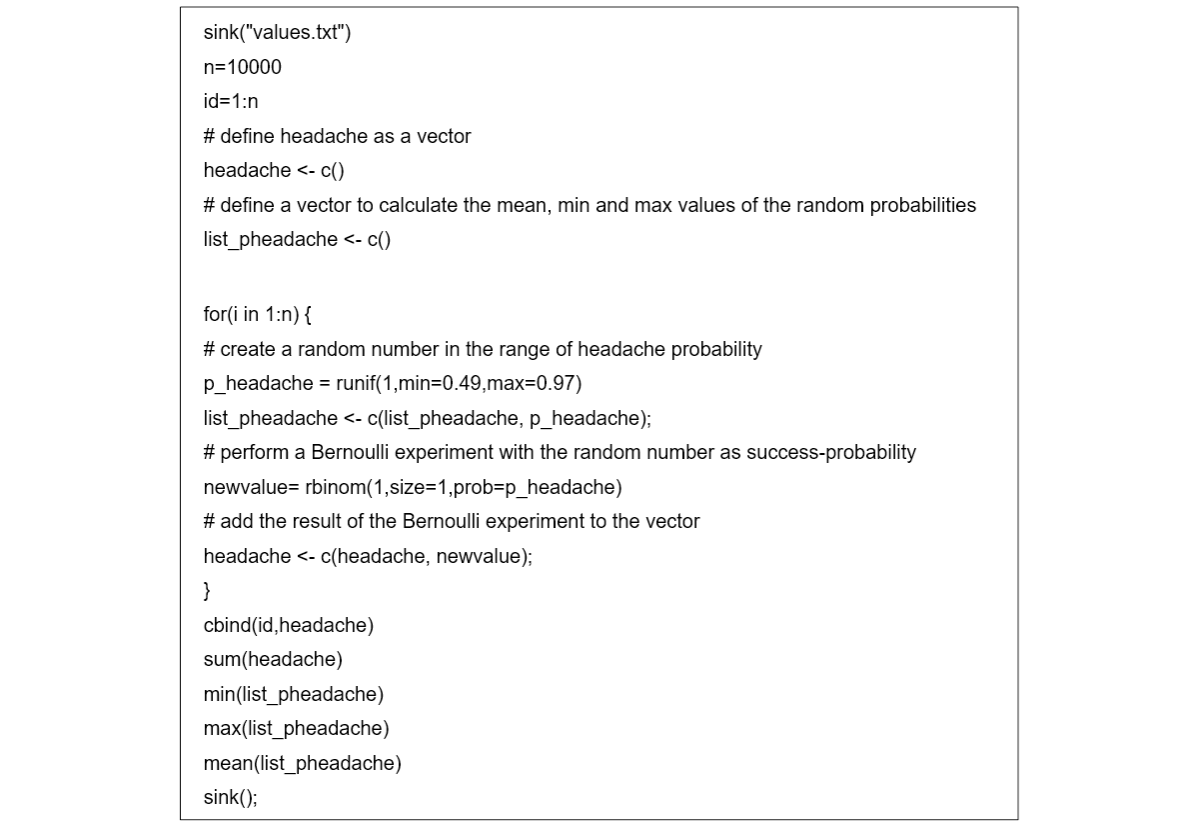
Example R script for the random generation of 10,000 cases with the symptom headache. # denotes a comment. The other symptoms are generated equally.

Based on these data, an R script was implemented to randomly create sequences of symptoms representing possible patient cases. Details of the script are shown in Figure 1.

The series of Bernoulli experiments was first simulated 10,000 times, resulting in 10,000 cases. For each case, the probability of success from Table 2 for the corresponding symptom was used. Table 3 contains the outcomes of the Bernoulli experiments.

**Figure 2:**
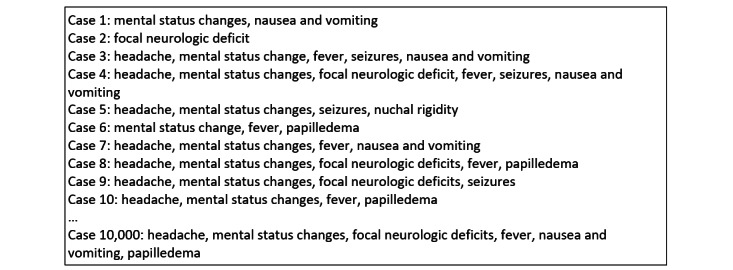
Virtual patient cases with respective symptoms.

For the generation of virtual patient cases, this means that, in case of success (outcome “1”), the corresponding symptom in Table 3 is assigned to the case. This leads to the virtual patient cases depicted in Figure 2.

With an increasing number of Bernoulli experiments, the relative frequencies of success and the average probabilities correspond more and more to the mean value of the range of probabilities from the literature (see Table 2). For example, the relative frequency for headache—7267/10,000 = 0.7267 (1. run)—rounded, is equal to the mean value of the range of the probability from the literature of 0.73.

Summing after 10,000 runs, this yields exactly the same ranges for the randomly drawn probabilities as in the literature.

The run of the R script can be repeated several times, with comparable results, as shown in Table 4.

**Table 3:** Outcomes of performing 10,000 series of Bernoulli experiments for “brain abscess.”

Patient case	Headache	Mental status changes	Focal neurologic deficit	Fever	Seizures	Nausea and vomiting	Nuchal rigidity	Papilledema
1	0	1	0	0	0	1	0	0
2	0	0	1	0	0	0	0	0
3	1	1	0	1	1	1	0	0
4	1	1	1	1	1	1	0	0
5	1	1	0	0	1	0	1	0
6	0	1	0	1	0	0	0	1
7	1	1	0	1	0	1	0	0
8	1	1	1	1	0	1	0	1
9	1	1	1	0	1	0	0	0
10	1	1	0	1	0	1	0	1
...	...	...	...	...	...	...	...	...
10,000	1	1	1	1	0	1	0	1
Sum of success	7267	5909	4271	5573	2396	5604	2889	2983
Estimated probability	0.7267	0.5909	0.4271	0.5573	0.2396	0.5604	0.2889	0.2983

**Table 4:** Comparison of 2 runs of the generator with 10,000 repetitions each, by showing relative frequencies.

Number of the run	Headache	Mental status change	Focal neurologic deficit	Fever	Seizures	Nausea and vomiting	Nuchal rigidity	Papilledema
1.	0.7267	0.5909	0.4271	0.5573	0.2396	0.5604	0.2889	0.2983
2.	0.7320	0.5897	0.4309	0.5515	0.2352	0.5604	0.2843	0.2961

## Discussion

### Principal Findings

In this work, we present a random number generator to generate virtual patient cases for a rare but fatal disease, for which missed diagnosis is an important prognostic factor [32]. The medical literature provides information on diseases with the associated spectrum of symptoms and the respective probability of occurrence of each symptom [20]. Using brain abscess as an example, a Bernoulli experiment was performed for each symptom with the probability of success based on the literature data. A series of experiments for the symptoms was started, and virtual patient cases with different symptom complexes were generated. We could show that the relative frequencies of the symptoms do not change significantly when the experiment is performed multiple times. The generator can create virtual patient cases at each start-up, which are different in their symptoms and, although these are random, they reflect the evidence-based probabilities from the medical textbooks.

A similar approach to ours using Bayesian networks has been applied to generate synthetic health data from real-world data in the field of heart disease and diabetes [33]. The external validity of the latter depends on the underlying sample, which is why we chose to use evidence-based basic information from the medical literature in our approach. However, a combined strategy may deliver the most realistic scenario.

### Limitations and Strengths

The main limitation of our generator so far is that specific symptoms are not sufficient to characterize a patient case. Additional information must be provided, and this should include, for example, the following aspects: age, gender, origin, socioeconomic aspects, further diagnoses, further symptoms, risk factors, or predisposing conditions.

The strength of our work is the compilation of evidence-based information into a template for full virtual patient cases. Our generator could build the basis of a program that helps medical teachers to provide cases of rare but fatal diseases in order to train and improve their student’s knowledge and skills in this regard. Furthermore, a larger number of distinct virtual patient cases could be made available and provide students with elaborated training possibilities.

### Future Possibilities

In our literature research, we were able to find information on several of these aspects [24,29-31], and medical textbooks are also rich with specific information that could be implemented in an automated generation of patient cases [19,20]. Our further literature research revealed that brain abscess, for instance, occurs more frequently in men (0.7/100.000) [34] and worse outcome is independently associated with Glasgow Coma Score on admission [35,36]. Hence, as an example, our generator could be expanded to determine gender as well, including a new Bernoulli experiment with the probability of success being 0.7 for male gender. The information on gender can then be added to the constellation of symptoms.

A further development of our generator can consider some of these other aspects in which patients differ. It would be of great benefit if a patient case with additional diagnostic criteria could be generated as a basic construct that would facilitate further elaboration. In the case of brain abscess, information on a predisposing condition like otitis media, sinusitis, or heart disease would be desirable. These conditions, together with the range of their relative occurrence, can also be found in the literature [34]. Moreover, a virtual patient should include laboratory data and media (like CT or MRI images), where necessary, as well as expert comments in the form of additional medical knowledge on a specific topic. For example, if there is a virtual patient with a suspected brain abscess, the expert comment “MRI is the first imaging choice for a patient with a suspected brain abscess. A lumbar puncture should be performed with caution only when there is clinical suspicion of meningitis or abscess rupture” could be given according to the literature [24,37]. It is further possible that medical information is needed not only in binary (true/false) form but also in a quantitative form with numerical values. For example, for the symptom “fever,” in some medical contexts, the numerical value is needed (eg, 38.5 °C). If this information is required, the authors of virtual patients would have to add the value manually. However, for known distributions or ranges, methods of random generation of data can also be applied. In addition, even conditional probabilities could be simulated within and under control of the program.

So far, the generator presented here does not provide any further information, and manual editing of the generated patient case is necessary to add it. A more elaborated version of our generator could provide an extended construct that saves medical authors’ time, which they can use in their clinical work, but it does not yet create a complete virtual patient.

Virtual patients and virtual cases are an integral part of medical teaching, especially in e-learning systems, but their development is expensive and complex [7,11]. Often, virtual patients are based on real patient histories that are prepared for use in scenarios that are also virtual [11]. Little is known about the automated generation of virtual patient cases, and using statistical distributions of patient or disease characteristics seems to be a completely new field. Instead of using data from single real patients, we used statistical information on aggregated data as they are presented in textbooks or epidemiologic surveys. In this work, we could take a first step in this direction and show that it is possible to generate virtual training cases by performing Bernoulli experiments based on probabilities from the literature. Hence, we could show that research in this new field is possible and should be further expanded. This can be a useful benefit, as medical staff, respectively medical teachers, are very busy, and the automated creation of virtual patient cases saves them time. As a result, medical teachers can spend more time with their real patients, and more virtual training cases are available. Furthermore, a shortage of cases of especially rare diseases can be avoided. In a continuation of this work, better-elaborated virtual training cases can be made available. This means that a constellation of symptoms and other data about a particular disease are presented, and the medical teachers can manually insert them into a virtual patient by adding further aspects such as expert comments, media, and feedback. As a result, the education of medical students can be improved.

### Conclusions

The results suggest that automated creation of virtual patient cases with rare diseases is possible, but with regard to the limitation of symptom constellations, it is not yet suitable for professional use. Our literature search showed that, for our exemplary rare disease “brain abscess,” a plethora of information can be found in the medical literature that completes the information found in conventional textbooks. Based on this additional information, an extension of the generator can be implemented in further research. In addition to the symptoms, all criteria with given probabilities can be transferred to the generation of virtual patients using further Bernoulli experiments. Other diagnostic criteria (eg, examination results) for which specific distributions are provided in the medical literature can be randomly determined by integrating different statistical functions into the generator. Virtual patient cases with more detailed clinical information are then generated by the random generator and can be provided to medical teachers and further elaborated as desired and may help training students in the diagnosis of rare diseases.

## Abbreviations

CT: computed tomography
MRI: magnetic resonance imaging
